# Barriers to using a helmet among motorcyclist students: a qualitative study

**DOI:** 10.5249/jivr.vo113i2.1543

**Published:** 2021-07

**Authors:** Vahid Ranaei, Zahra Hosseini, Sakineh Dadipoor

**Affiliations:** ^*a*^ Student Research Committee, Faculty of Health, Hormozgan University of Medical Sciences, Bandar Abbas, Iran.; ^*b*^ Associate professor of Health promotion and Education, Tobacco and Health Research Center, Hormozgan University of Medical Sciences, Bandar Abbas, Iran.; ^*c*^ Social Determinants in Health Promotion Research Center, Hormozgan Health Institute, Hormozgan University of Medical Sciences, Bandar Abbas, Iran.

**Keywords:** Road Accidents, Motorcyclists, Helmet Use, Effective Factors, Qualitative, Research

## Abstract

**Background:**

Helmet use rates among motorcyclists are low and various factors are involved. Therefore, the present study aimed to investigate the factors affecting the use of helmet in motorcycle students in Iran (Bandar Abbas city) in 2019.

**Methods:**

The research was qualitative and data were collected through individual interviews and observations and were interpreted by content analysis method. Participants were 15 motorcyclist students studying in public health (6), nursing (4), dentistry (2), medicine (1), health education (1) and biochemistry (1). They were purposefully included in the study and sampling continued until data saturation. The main tool for data collection was deep semi-structured interviews with open answers which lasted for 20 to 60 minutes. Finally, 15 interviews were collected in this study.

**Results:**

The five main categories (economic, family, socio-cultural, individual, and riding rules) were extracted from the data that each had a subclass.

**Conclusions:**

Different factors in micro and macro dimensions play a role in the use of helmets among motorcyclists. Consideration of these factors by the relevant organizations in the field of traffic can increase the use of helmets.

## Introduction

Regarding the World Health Organization (WHO) in 2018, the annual number of road casualties has reached 1.35 million and about 3,700 people are killed in road accidents around the world every day, which these accidents include cars, buses, motorcycles, bicycles, trucks and pedestrians.^1^ In addition, road traffic injuries are currently the leading cause of death for people aged 5 to 29, especially those living in developing countries and more than half of those killed were pedestrians, motorcyclists and cyclists.^1^

In fact, motorcycle injuries are a major and increasing public health problem worldwide.^2^ Statistics show a significant increase in motorcycle casualties in recent years. With an increase of 48%, from 3,365 cases in 2002 to 4,976 cases in 2015.^3^ 23% of riding casualties in the world occur among motorcyclists while this rate is 23.8% in Iran.^4^ Meanwhile, the share of motorcycle casualties is significant in comparison with the total casualties of traffic accidents in Golestan and Hormozgan provinces.

Motorcycles are less stable and less visible than other vehicles.^5^ Hence, the rate of accidents for motorcycles is four times higher than for passenger cars.^6^ Due to the lack of safety equipment such as seat belts, lower limb protectors and airbags than other passenger vehicles, motorcyclists who crash are severely injured. As a result, motorcyclists are three times more likely to be injured than car occupants, and 16 times more likely to die if they fall or crash.^7^ Head injuries are the most common injuries to motorcyclists,^8^ and the use of helmets can reduce the risk of head injury. Therefore, reports show that mortality and disability are lower in motorcyclists who use helmets.^9^ However, most motorcyclists do not use helmets.^10^ For example, in a study by Hassan et al.^2^, only 7% of motorcyclists with injuries caused by accidents had worn helmets.

In addition, it is difficult to change the habits of motorcyclists in societies that have not used helmets from the beginning. In these societies, the norms are not established for the use of helmets, and many people know this traffic behavior as an unusual and unnecessary behavior. For this reason, the status of individual norms and beliefs in these societies can be one of the main barriers in the use of helmets by motorcyclists.^11,12^

In Iran, the Traffic Law has banned motorcycles from riding without helmets since 2001, and has required law enforcement officers to enforce the law on riders who do not use helmets. However, the available statistics indicate that the rate of helmet use in Iran is low.^13^ The rate of helmet use in Iran among motorcycle riders is estimated at only about 30 percent and for other occupants at 10 percent.^14^

Overall, despite the inefficiency of motorcycles in providing safety, it is still an economical vehicle for a large portion of the people, especially in cities with high traffic congestion and inefficiencies in public transportation.^8^ In addition, given the Iranian government's approach in eliminating gasoline subsidies, the use of motorcycles is expected to be increased, especially in large cities. Therefore, the above points emphasize the need for qualitative research in this field.

According to the results of studies, the most important reasons for not using a helmet are the lack of comfort, limited vision and inability to control the path, feeling of sweating during use, feeling of heat in the head and getting tired.^15^ But it is possible that the use of helmets is affected by other factors in addition to those previously mentioned. According to the contents of this qualitative research, the aim of determining the factors affecting the use of helmets was done through interviews with motorcyclists in Bandar Abbas city.

## Methods 

This research was conducted qualitatively and by content analysis method. Content analysis method has some strengths and weaknesses. Content analysis is a flexible method and can be used for a wide range of textual data. The existence of computer software makes it possible to analyze large amounts of data by content analysis. It can be used to analyze data that has occurred naturally. It can be used to study a topic longitudinally by analyzing related resources over time. It can also reduce social desirability bias among participants when researching sensitive topics. Difficulties related to sampling and coding can be associated with bias in content analysis. In addition, paying attention to a word or sentence separate from its original text can lead to misunderstanding.^16^ In this study, because the researcher sought to investigate the factors affecting the use of helmets among motorcycling students, he used a qualitative method (semi-structured interviews) and content analysis. Like this, far from using any presuppositions and without intermediaries, students' reasons should be extracted from their own point of view.

***The participants***

The participants were motorcyclists of Hormozgan University of Medical Sciences in the academic year of 2009. Fifteen of them participated in the study using purposeful sampling method: 6 in the field of public health, 4 in nursing, 2 in dentistry, 1 in medicine, 1 in health education and 1 in biochemistry. These were people who had enough experience in the subject matter. Participants in this study were selected from different age groups, different educational levels, different academic disciplines, including single and married to achieve maximum diversity.

***Data collection***

In-depth and semi-structured interviews were conducted with open-ended questions, and participants were given the opportunity to describe their experiences on motorcycling.

In all interviews, the interviewer introduced himself firstly and, after explaining the purpose of the study, provided the participant with a consent form to study and complete. After the initial conversations and thanking the person for participating in the research, the interview began with these questions:

What experiences have you had with motorcycling? What steps do you take to maintain your safety while riding a motorcycle? 

The rest of the questions were based on the participants' responses to these questions.

Questions such as "What exactly do you mean?" or "Would you please explain in more detail? The interviews duration was about 20 to 60 min based on the comfort and the willingness of the participants. The voices were recorded after taking participants consent.

***Data analysis***

Conventional content analysis was used to analyze the data. In such a way that, the recorded contents were implemented and copied word by word after ending interviews and group discussions. After doing each interview and the content inclusion to the mentioned software, the interviews contents were read line by line and each segment of the participant speech related to the research question was coded. The codes were classified and compared based on the similarities and differences between codes and classes in all interviews.

***Validity and Reliability***

Continued relationship with participants in the research environment, sufficient time to data collection, supervisors' survey and using member checking were considered for data validity and reliability.

## Results

As shown in Table 1, Most of participants were studying for bachelor degree (66.6%) and in public health (40%). Most of them were single (73.3%) and 60% were living in the village. 

**Table 1:** Demographic status of motorcyclists.

Variable		Motor cyclist students	
		Frequency	%
**Sedentary location**	City	6	40
	Village	9	60
**Marital statue**	Single	11	73.3
	Married	4	26.6
**Academic course**	Bachelor	10	66.6
	Master	2	13.3
	Public PhD	3	20
**Field of study**	Public health	6	40
	Nursing	4	26.6
	Health education	1	6.66
	Biochemistry	1	6.66
	Medicine	1	6.66
	Dentistry	2	13.3

Five main subjects of economic, family, sociocultural and individual factors and traffic laws and 17 subclasses were obtained based on the participants' views on the factors affecting the use of helmets in motorcyclists (Table 2).

**Table 2:** The factors affecting the use of helmets in motorcyclists

Main Topic	Sub-topic
**Economic factors**	1. High price and low quality helmets
	2. Family expenses provision
	3. High cost of treatment due to the effects of the accident
	4. Imposing financial burden on the government
**Family factors**	1. Family support
	2. The value of health and welfare
	3. Commitment to family
	4. To be ridiculed
**Sociocultural factors**	1. Observational learning
	2. Mobile fever
	3. Demonstrational relief
**Individual factors**	1. Injury experience
	2. Fear of death
**Traffic laws**	1. Disregard for the law
	2. Fear of being fined
	3. Believe in law effectiveness
	4. Vehicle detention

1. Economic factors: this class includes factors related to the income statue of participants. Four subclasses were recognized including: the price and quality of helmet, providing family expenditures, the costs of accident treatments and imposing financial burden on the government. Sub-topics are as follows:

High price and low quality of helmet: participants believe that high price helmet is effective in not using it. "With this initial cost of buying a hat that we want to accept, the quality of the hat is very low." (Participant No. 2). 

Providing family expenses: Some participants cited the use of helmets as a reason to protect themselves and provide their families expenses. “It is better to use a helmet and keep myself away from accidents because I am the breadwinner of my family and I should not have financial problems” (Participant No. 11).

Accident treatment costs: Participants cited the cost of treatment and prevention and disabilities as effective factors in using helmets and preventing traffic accidents. "When you wear a helmet, if you have an accident, there will be no damage to your brain, and when you have an accident, the cost of hospitalization will be much lower" (Participant No. 1).

Imposing financial burden on the government: Participants also saw the creation of a financial burden on the government and society as an effective factor in the use of helmets, stating that "it may make sense for me to wear a hat, not a cost to my pocket and regarding what they say on TV, I don't impose a financial burden on the nation and the government” (Participant No. 4).

2. Family factors: This category includes issues related to maintaining the institution of the family and the responsibility of the individual towards family members. The four sub-topics identified in this group are family support, value of health and welfare, and commitment to the family.

Family support: From the view of the participants in the study, the advice of the spouse and the emphasis of the parents have a significant effect on the use of helmets. "My wife emphasizes that if you don't get used to it now and don't wear a helmet, your health may be affected" (Participant No. 7).

The value of health and welfare: Another factor that played a significant role in the use of helmets was the commitment of motorcyclists to their spouses and parents and the comfort of family members. "Sometimes it scares me that my youth is in danger, it's not only because of myself, it's more because of my family" (Participant No. 10).

Commitment to the family: Participants in the study believed to maintain their health by observing safety tips for being a healthy parent in the future in any situation. "Sometimes it scares me that my youth is in danger, not so much because of myself, but more because of my family and the future of my children" (Participant No. 5).

To be ridiculed: One of the most important barriers for motorcyclists to not wear helmets was being ridiculed by family members. "When my brother is with me, I don't like to wear a helmet for motorcycling, he always says these things are for inexperienced people and he laughs at me" (Participant No. 12).

3. Socio-cultural factors: This class includes modeling and the influence of other people in society on learning behaviors. Sub-topics were: observational learning, mobile fever, and demonstrational relief.

Observational learning: In this study, participants believed that the use of helmets by others had a significant effect on their use of helmets. "When you see that everyone is obeying the rules, you are doing the same, but when you see that many are not doing and nothing is happening, you are not wearing a helmet" (Participant No. 12).

Mobile fever: One of the most important reasons why motorcyclists do not use helmets was their need to use a mobile phone while riding a motorcycle. "I often have to be available when riding a motorcycle and my phone rings too much and my friends or loved ones call" (Participant No. 9).

Demonstrational relief: Seeing the experiences of other people affected by not wearing a helmet is effective in using a helmet. "In front of me, a man had an accident and his head was injured, and because he was an acquaintance, I saw that his family was affected. That's why I wear hats regularly for three to four weeks” (Participant No. 15).

4. Individual factors: This class included the person's experiences and feelings about wearing a helmet, which included two sub-topics of having the experience of injury and fear of death.

Injury experience: According to motorcyclists, having a car accident experience is a factor in using a helmet. “I had an accident experience myself, if I had a helmet I wouldn't have been injured and I wouldn't have had to rest for so long" (Participant No. 14).

Fear of death: From the view of participants, the fear of death persuades motorcyclists to wear hats. "I leave the helmet because to be honest, I'm a little scared to death" (Participant No. 8).

5. Traffic laws: This category includes traffic laws for the use of helmets, and its sub-topics include disregard for the rules, fear of being fined, and belief in effectiveness of laws and confiscation of vehicles.

Disregarding the laws: disregarding the rules prevents the use of helmets while riding motorcycles. "In the issue of motorcycles, I don't really believe in helmets and I don't use it" (Participant No. 6).

Fear of being fined: According to the participants in the study, the view of the police and those around them and the fine for not wearing a helmet will lead to follow the rules of traffic. "I have to use it because the police or the acquaintances are watching, and it may not be right for me to violate the rules in front of the acquaintances" (Participant No. 3).

Belief in the effectiveness of rules: Participants believed that traffic rules had no effect on the number of accidents. "Maybe the fines they impose aren't very practical, and I don't really believe in fines myself."

The confiscation of vehicles: from the participants' views, transferring the motorcycle to the parking will lead to use helmet while riding. "My car was damaged and taken to the parking. So I traveled for two or three days and was suspended" (Participant No.13).

## Discussion

The aim of the study is assessing the factors effective in using helmets among motorcyclist students in Bandar Abbas city. It was seen in the study that economic factors are among the factors effective in using helmets among motorcyclists. The price and quality of helmet, family expenses provision, the costs of accident treatment and imposing financial burden to the government are the economic factors cited by the study participants effective in using helmet. In the study by Arjoui et al. (2014), the high price of helmet is mentioned as one of the reasons for not using it.^17^ Indeed, when the quality of helmet is not proportional to its cost, the people are not willing to buy it. Because unsafe helmets will lead to more damages than the standard ones.^18^ Furthermore, reducing the rate of road accidents can affect Gross Domestic Product (GDP) by 1-3 percent.^19^

Family factors are also effective factors in using helmets. Family support, the value of health and welfare and commitment to the family are among the factors related to the family. In line with this, the study of Saridhahran et al. (2010) also showed that being married is related significantly to using helmet.^20^ Although, the effect of family factors in using helmet is not approved in studying the history of another qualitative study, we must mention that motorcycle is a common vehicle in the families of majority of low and mean income countries especially in Asian countries.^21^ Therefore, road accidents as an increasing concern in the field of health issue leads to many material and immaterial losses in victim families.^19^

Another class of effective factors includes sociocultural factors consisted of observational learning, mobile fever and demonstrational relief. Many studies were obtained which were correspond with current study findings and emphasized on the effective role of culturalization, teaching and informing and its effect on the social norms in using helmet.^17,13^ So, media mainly TV as a convincing tool can be effective in increasing the use of helmet.^22^ In addition, it was shown that social meetings aiming at increasing knowledge and culturaliztion to meet safe behaviors while riding motorcycle, are effective in increasing the rate of using helmet.^23^

Individual factors including fear of death and damage experience can also affect the use of helmet. The study of Koolantayan et al showed that having prior experience of accident is effective in using helmet.^24^ Indeed, far from outside encouraging or punishing and with a higher level of authority and choice, the people resort to wearing helmets when they face individually with the accident scene and its results. Studies have also shown that when people digest the advantages and disadvantages of behaviors (identification motivation), they are more willing to do the desired behavior.^25^

In the end, the factors related to traffic laws such as disregard for the law, fear of fines, belief in the effectiveness of the laws, and vehicle seizures were all effective in using helmets. In line with this finding in the present study, studies have shown that the use of helmets is more common in areas under police surveillance.^26^ And less understanding of the dangers and less likely to be enforced by traffic officials is associated with less compliance with road safety rules and regulations.^27^ Also, in another study, the majority of motorcyclists reported that restrictions of the law and police were effective in using helmets, and that if police did not inspect and enforce the law, most of them would not wear helmets.^19^

This study was also associated with limitations. It should be noted that the research method in the present study is qualitative, which although identified some factors affecting the use of helmets, but is unable to investigate the causal relationship between these factors and the behavior of helmet use. Therefore, future studies can examine the causal relationship between these factors with using helmets in experimental study designs. Since this study is based on survey data and in fact some of the important variables might have not been collected. In fact, there could be still some unobserved factors which could influence helmet use but might not be covered in the survey. Also, the sample studied in the present study was motorcycle students in the city of Bandar Abbas, which limits the generalization of the findings to the motorcycling community of the country. However, conducting in-depth interviews with students provided a comprehensive understanding of the reasons why they did not wear helmets. It was observed that a wide range of micro and macro causes are effective in this field and intervention measures in this field should consider all aspects. Therefore, the findings of this study can provide guidance for relevant organizations and institutions in the field of traffic to design and implement programs to reduce the rate of injuries caused by road accidents, especially traffic accidents related to motorcyclists.
